# Monitoring of Temperature in Retail Refrigerated Cabinets Applying IoT Over Open-Source Hardware and Software

**DOI:** 10.3390/s20030846

**Published:** 2020-02-05

**Authors:** José Ramírez-Faz, Luis Manuel Fernández-Ahumada, Elvira Fernández-Ahumada, Rafael López-Luque

**Affiliations:** 1 Department of Electrical Engineering, University of Córdoba, Campus de Rabanales, 14071 Córdoba, Spain; jramirez@uco.es; 2 Department of Computing and Numeric Analysis, University of Córdoba, Campus de Rabanales, 14071 Córdoba, Spain; 3 Department of Mathematics, University of Córdoba, c/San Alberto Magno s/n, 14071 Córdoba, Spain; elvira@uco.es; 4 Department of Applied Physics, University of Córdoba, Campus de Rabanales, 14071 Córdoba, Spain; fa1lolur@uco.es

**Keywords:** retail cabinet refrigeration, temperature control, IoT, open-source hardware and software, cloud computing

## Abstract

The control of refrigeration in the food chain is fundamental at all stages, with special emphasis on the retail stage. The implementation of information and communication technologies (IoT, open-source hardware and software, cloud computing, etc.) is representing a revolution in the operational paradigm of food control. This paper presents a low-cost IoT solution, based on free hardware and software, for monitoring the temperature in refrigerated retail cabinets. Specifically, the use of the ESP-8266-Wi-Fi microcontroller with DS18B20 temperature sensors is proposed. The ThingSpeak IoT platform is used to store and process data in the cloud. The solution presented is robust, affordable, and flexible, allowing to extend the scope of supervising other relevant parameters in the operating process (light control, energy efficiency, consumer presence, etc.).

## 1. Introduction

The agrifood chain is facing significant new challenges nowadays. Among others, monitoring and controlling temperature along supply chains emerges as a key aspect to deal with food waste [1,2,3], as well as increasing both food safety and the quality offered to consumers [4,5,6].

Whereas, the early stages of processing and distribution compliance with the temperatures established for food safety was reached [6], however, in the last three stages (considering here transport, retail, and households), temperature control and maintenance have become particularly complex [4,7,8]. In the retail sector in particular, the scarcity of data available on the fulfilment of cold chain control is highlighted [9,10]. In addition, there are many studies confirming that the temperature of display cabinets in refrigeration units is not always the appropriate one, according to safety standards [11,12,13,14].

In Europe, there is a regulation by the European Council and Parliament assigning food companies the responsibility for complying with temperature control requirements and microbiological criteria applicable to food products, as well as cold chain maintenance [15]. Concerning this last aspect, the regulation establishes the importance of controlling temperatures and proper operation of refrigeration equipment, considering that daily temperature reading is a valid method to control the cold chain in retail establishments. However, there are studies proving that this method cannot guarantee compliance with the perishable food safety specifications, especially in southern Spain [9].

Among the technologies offering temperature control solutions currently available throughout the cold chain, wireless sensor surveillance technologies, especially radio frequency (RF) and wireless sensor networks (WSN), have been regarded as leaders in this field [16,17], with many works on its implementation to achieve global food temperature traceability at different points in the chain, becoming part of broader food safety systems [18,19,20,21,22,23]. Nevertheless, far fewer research studies in the retail sector refer to cabinet and refrigeration equipment temperature control. Most of the scientific literature focusing on the retail sector emphasizes the estimation of the shelf life of food located in refrigeration equipment or whether they keep the required temperature levels [14], yet avoiding a deeper study of the systems used for temperature measurement and monitoring.

In order to provide solutions to solve this challenge, Internet of things (IoT) systems are becoming more relevant, where a wide range of sensors are connected to each other along the cold chain, sending useful information for decision-making and preventing potentially undesirable events [16]. As a result, the IoT is expected to help retail chains to control the quality of food products, to plan the handling of end-of-life waste, to manage the temperature of freezers, refrigerators and warehouses, and to contribute to the reduction of energy consumption [24].

On a commercial level, there are large companies specialised in refrigeration equipment [25,26], which offer systems for monitoring temperature, but which have the disadvantage of being expensive and do not offer the possibility of easily accessing the data generated or being integrated into systems that monitor other types of parameters. Other options, based on data recording devices located in each refrigeration unit [27,28], offer a cheaper alternative, but without constituting an affordable solution for small and medium-sized companies, for which this term is critical [29].

This is the line in which the proposal presented in this work is situated in order to respond to the challenge of monitoring the temperature of refrigeration equipment in the retail sector, by using new trends in technological development, such as free and open-source hardware (FOSH). FOSH is a hardware whose design is made publicly available so that anyone can study, modify, distribute, make, and sell the design or hardware based on that design [30].

In view of the above, the aim of this research is to develop a low-cost IoT system, based on free hardware and software, for monitoring the temperature in refrigerated retail cabinets. This system provides solutions in domains such as price management in perishable products, the frequency of temperature measurement in retail establishments, and economic problems caused by food waste [9,16,31]. Another aim is to create a system with enough flexibility to extend the scope of supervising other relevant parameters in the working environment (door opening-closing, consumer presence time in front of the exhibitor, lighting, and energy consumption).

The current work shows a complete functional solution for temperature monitoring in the food cold chain. By proposing it for open access publication and by developing it as open-source software and hardware, it confers an innovative aspect to the set of solutions available in the market. Thus, the full accessibility to all the details about the designed device opens the possibility to communities of scientists and technicians to correct and improve aspects, extend functionalities, replace components, or compare operation modes.

Nowadays, the emergence of freely participating communities in collaborative projects with scientific-technical objectives with free and accessible information is recognized as a revolution of the scientific development paradigm. In this sense, the work presented, developed as the genesis of a collaborative project, is a pioneer in proposing an open solution that is already operational, but which can still be improved by the scientific community. Undoubtedly, these improvements will contribute to the generation of scientific knowledge as well as to the improvement of humankind’s access to food.

The next section discusses the alternatives available in this research field and is followed by the solution design. The paper then focuses on the experimental implementation and solution evaluation. Finally, conclusions and research lines of future work are presented.

## 2. Related Work and Technical Background

Temperature monitoring within the food distribution sector is supported by different technologies for information transmission, processing and control, measurement, and remote data storage and management.

As far as the information transmission is concerned, the use of wireless systems (WSN) have become widespread in opposition to wired systems that are expensive and not very flexible for scaling [32,33].

**Figure 1:**
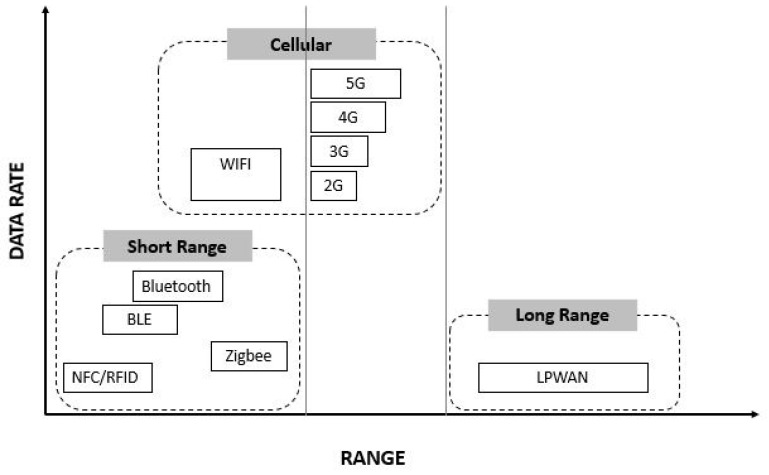
Data rate versus range in wireless networks. Source: [35].

To provide support to WSN, communication networks are required. Three network clusters classified by range and transmission speed are identified [34]: (i) short-range networks, (ii) cellular networks, and (iii) long-range networks (Figure 1).

Short-range networks, notably Zigbee or Bluetooth, have no licence to use. Among their features (Table 1), low power consumption and high exchange speed are the most remarkable. Research works used Zigbee devices to monitor cold rooms and estimate energy consumption and product water loss [36] or vegetable transportation under real work conditions [37]. The work using Zigbee devices, analysing battery behaviour as well as data exchange quality for the control of refrigerated products, is also worth mentioning [38].

Still in the field of short-range networks, there are numerous applications based on radio frequency identification (RFID) covering areas ranging from shelf life control [39], through live animal transport control [40], and environmental variable monitoring such as humidity, temperature, and volatile gases. [41]. Applications with near-field communication (NFC) are less frequent than those based on RFID, but their compatibility with smartphones and tablets provide an interesting niche market. To this end, an application based on NFC sensors controlling meat product traceability have been developed [42].

Furthermore, cellular networks enable a high transmission speed in both short range (Wi-Fi) and long range with license (GSM, GPRS, 3G, 4G, and 5G). There are examples of applications of cellular networks that use GPRS communication in sample shipments from refrigerated trucks to generate shelf-life predictions for transported products [21]. In another work, the authors employ Wi-Fi and GPRS-compatible architecture, among other networks, to monitor environmental variables of perishable food containers with a very low rate of data loss in communication [20].

In the field of long-range networks, low power wide area networks (LPWAN) are being developed, being compatible with the IoT concept for low transfer rates and having lower energy consumption versus cellular networks (2G, 3G, 4G, and 5G) [43].

The free hardware philosophy has been used to **control and process** data in a wide range of technological applications. These technologies have not been used in the food sector yet, but they are gradually becoming more and more popular due to boards equipped with Bluetooth, Wi-Fi, Ethernet, Lora, LORAWAN, SIGFOX, Android compatibility, and a catalogue of sensors designed to interact and measure any variables. In this scope, ATMEL microcontrollers and those compatible with its development environment (IDE) are relevant [44]. Its features (free philosophy) together with low cost and size have favoured a gradual incorporation into the food traceability sector [45], combined with Android devices for meat quality control of beef [46] or for the foam quality of sparkling wines [47].

Table 2 details the most widely distributed boards integrating this open source philosophy and offering an intelligent node between temperature data reception, management, and cloud interaction as they include modules for wireless communication.

Within the scope of measurements, temperature reading in retail systems is a basic element in any refrigeration system architecture. As a result, a study of the different sensors is required. A variety of temperature sensors can be identified [48]: Resistive, electronic, and electromechanical. Table 3 shows the characteristics of those most frequently used in industrial refrigeration.

Concerning cold chain management, every technology presented in Table 3 was applied. Both the variety of temperature ranges studied for different products and the importance of controlling temperature have been emphasized [49]. Research works using silicon sensors to monitor the temperature in warehouses, containers, and vehicles [20] and for drying control by means of infrared sensors [50] are remarkable.

**Figure 2:**
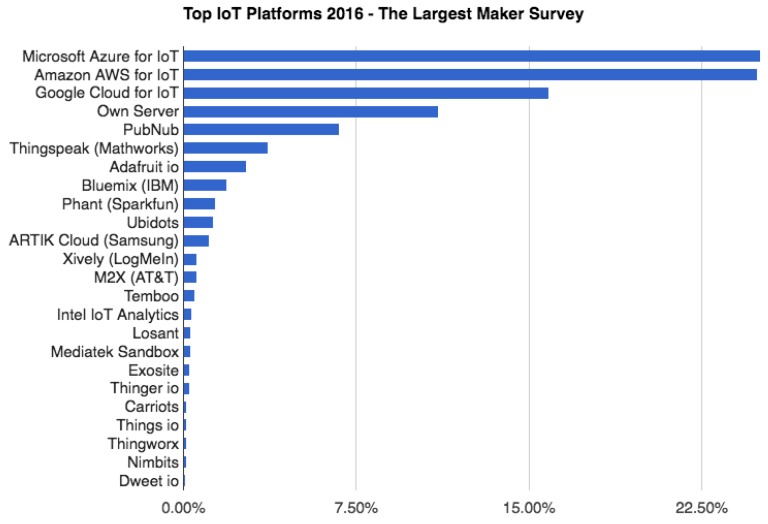
Top Internet of things (IoT) platforms. Source: [55].

Finally, the technological demand for remote data storage and management (monitoring and performance based on IoT and cloud computing) is addressed in three ways in the bibliography [51,52]. First of all, specific programming models for given problems are available. This method implies a considerable programming effort since it requires the whole application codification without code reuse or adaptation to standard software. Secondly, there are adjusted commercial solutions in terms of measuring and uploading data to the cloud. Within this scope there are systems like Stellapps, an end-to-end dairy technology solutions company, providing IoT, big data, and monitoring services in the daily fresh produce area [53] or Innovecs which offers remote cold chain control services monitoring and controlling significant variables [54]. These solutions are closed to the user which represents a significant drawback. Finally, another approach to these technological challenges is dealing with generic commercial tools (IoT platforms) where the developer is responsible for customising the application to suit the required needs. Figure 2 displays the most representative IoT platforms as well as their market distribution [55]. Dealing with these IoT platforms, it is worth highlighting a research work that developed an IoT-based route planning system [56] was based on the IBM platform [57], and the one that was developed a platform to virtualize food supply chain was based on FIspace [58], a cloud-based platform for business collaboration [59].

## 3. Design and Implementation of the Proposed Solution

This work deals with the development of a temperature monitoring system based on free hardware and software (see Table 4), applying the IoT concept. The proposed solution was applied in an establishment of a chain of 75 stores (Andalusia, Spain).

### 3.1. Architecture of the Solution

**Figure 3:**
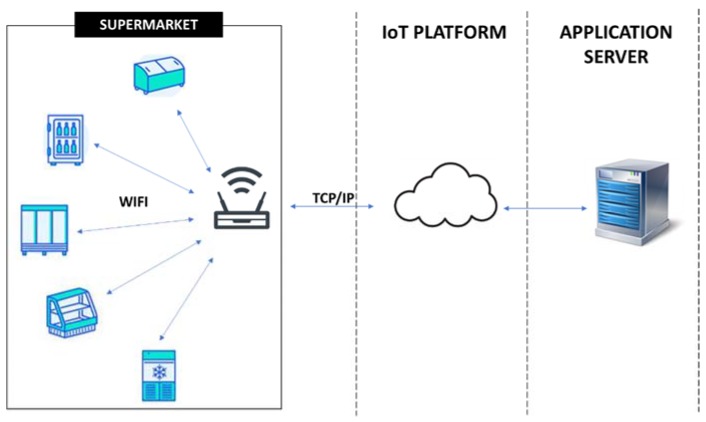
General solution based on Wi-Fi communication.

With regard to local architecture communication, considering the options detailed in Section 2 and due to the average size of the establishments where the proposed solution will be deployed, LPWAN was excluded because it is long-range. From the remaining options, Wi-Fi (cellular network) was chosen because it offers a higher speed than short-range solutions, its performance range is better suited to the size of an average-store and, essentially, most food establishments are equipped with Wi-Fi. Within the framework of a low-cost solution, it was considered appropriate to take advantage of the existing network for the exchange of information. Moreover, its bandwidth copes with the monitoring system requirements and this choice for device Internet access eliminates the need for a hub/gateway. Figure 3 shows how a general solution would be developed on the basis of Wi-Fi.

**Figure 4:**
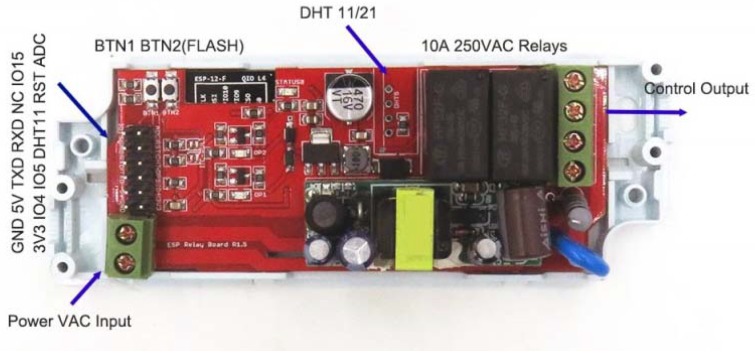
Electronic board Electrodragon ESP Relay.

Devices equipped with Wi-Fi communication for cloud exchange information were considered for the control and processing of temperature readings. The characteristics and cost of the boards analysed are presented in Table 2. In this project, the Electrodragon ESP Relay (Figure 4), based on the ESP-12F module equipped with the ESP8266 microcontroller, was chosen. Its price is the lowest among the boards considered. An essential feature of this board is that it is powered from the AC mains at 230 Vac, since it integrates a 230 Vac/3.3 Vdc power supply. It also has two relays with a switched contact for voltages up to 250 Vac and 10 A. This solution enhances options based on RFID and BLE (Bluetooth Low Energy) communications with battery-powered nodes [17], which dismiss the Wi-Fi solution allowing greater range by making the system developed incompatible with a stand-alone solution. Although there are other options with a greater number of inputs and outputs, this is enough for the development of the proposed solution. Studies in the literature [23] show that the number of inputs supported by the central unit is limited. In the proposed solution, the system is decentralized, achieving a system with control based on ESP8266 enabling multiple inputs at a very low cost.

Considering measurement scope, the DS18B20 was selected from among the sensors analysed (Table 3) due to its accuracy, measurement range, low cost, and communication standard and for its broad use within the food industry literature [60,61]. Table 5 shows the most relevant characteristics of this silicon sensor.

**Figure 5:**
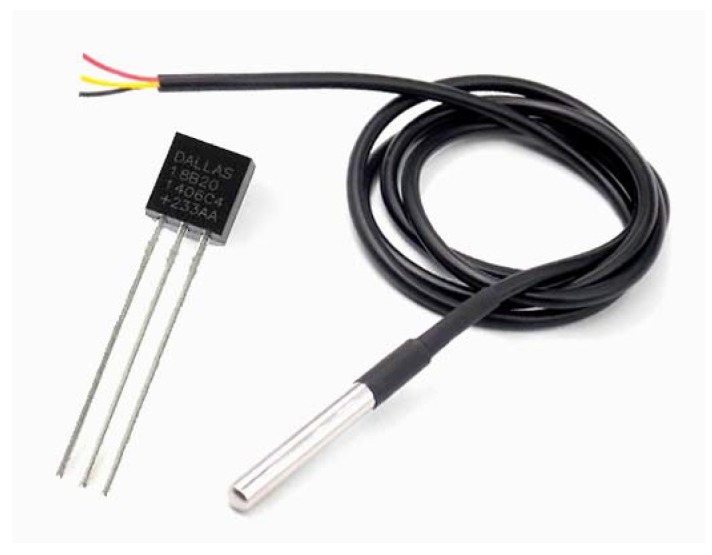
Sensor DS18B20 with TO-92 enclosure (left) or sealed stainless steel sheath (right).

The sensor is provided in TO-92 enclosure or sealed stainless steel sheath (Figure 5). This second option was chosen to allow immersion in water. Each sensor has an identification number engraved on its ROM, composed of 64 bits.

**Figure 6:**
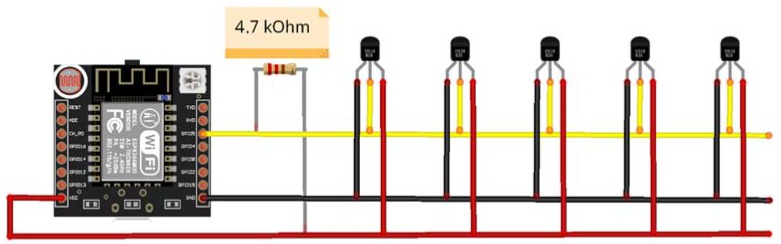
1-wire bus scheme.

Connections between the electronic board and temperature sensors were established with the communication protocol 1 wire, a master slave type, developed by Dallas Semiconductor. For a maximum distance of 25 m up to 100 slaves (sensors) can be connected to the bus by implementing a 4.7 kΩ pull-up resistance. The bus consists of three wires: Vcc, GND, and DATA. In this way, with a microcontroller it is possible to monitor the temperatures of a large part of the equipment of an establishment. Although, given the cost of the microcontroller, it may be more profitable to increase the number of these as opposed to the extension of the bus wiring to the different measuring points. Figure 6 shows the connection diagram of the sensors.

In cases where a single sensor is connected such as 1-wire bus protocol, it is not necessary to specify the direction of the sensor.

**Figure 7:**
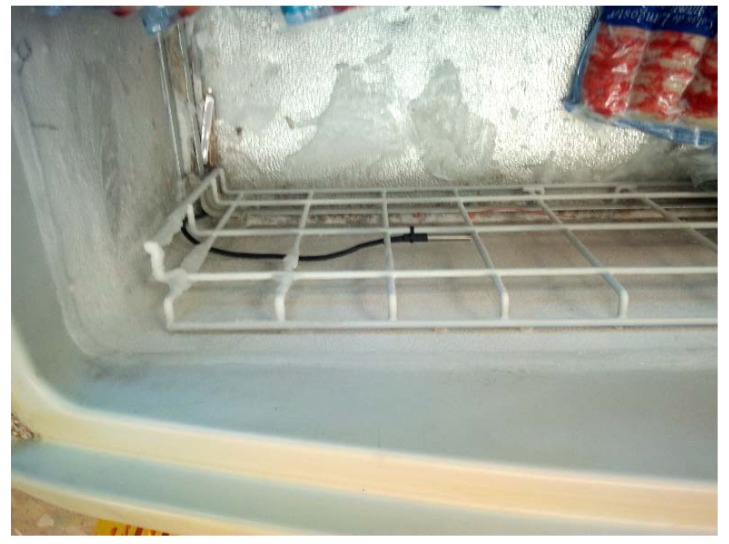
DS18B20 probe placed in a freezer for the experimental setup.

Implementation in the field is simple, placing the temperature probe (DS18B20) next to the one corresponding to the control thermostat in the chilling cabinets. Considering that the probes are wired up to the board and, since the size of the average store is within the working range of the Wi-Fi network, it is not necessary to adopt a methodology for the deployment of sensors that would be mandatory in other types of applications with limited coverage [63]. The control board (ESP8266) takes the electrical supply from the same source as the cabinet. The board ESP8266 has a very low energy consumption (0.3 W average), so it can be connected to the cabinet power supply without affecting the normal operation of the cabinet and the electrical protections. The solution described avoids the use of batteries, which would imply a higher maintenance cost. Figure 7 shows the probe location in the freezer used for the experimental device. In a commercial solution, protection against intentional damage would be necessary.

Once all components are connected, programming can be implemented. There are libraries facilitating the use of this sensor on boards supporting the Arduino IDE for programming. In our proposal DallasTemperature.h and OneWire.h, and the ESP8266Wifi.h libraries were utilised [44].

**Figure 8:**
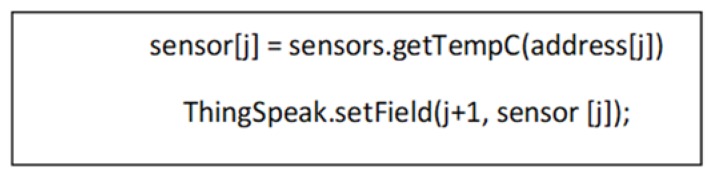
Reading and delivery of a temperature sensor.

Finally, the solution requires the choice of a system for remote data storage and management. As highlighted in Section 2, alternatives based on specific programmed models and closed commercial solutions had many drawbacks. In the present case, a solution based on an IoT platform was chosen by customizing our specific application. Concretely, the educational version of the ThingSpeak platform was used [64], which provides up to four channels with eight fields each, thus 32 variables can be updated at intervals of 15 s per channel. It supports up to three million data per year in this type of non-commercial account. The possibility of inserting the information in different ways is contemplated: HTTP, MQTT, and API-REST. In this project we opted for HTTP, using the ThingSpeak.h library for the Arduino IDE. The use of this library simplifies the writing procedure in the ThingSpeak channels (Figure 8).

The platform stores the information transmitted according to the structure described. Additionally, this platform provides some mathematical analysis tools based on MATLAB highlighting statistics and machine learning toolbox, deep learning toolbox, and predictive maintenance toolbox.

**Figure 9:**
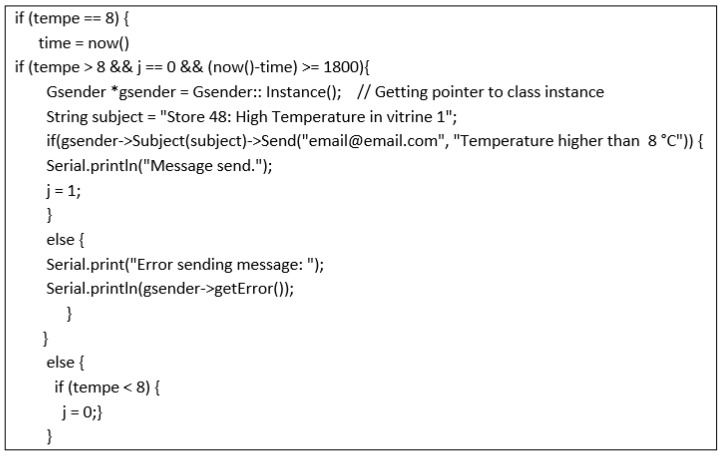
Alarm generation code.

Communication between each ESP8266 board and the ThingSpeak platform was made via the WLAN of the establishment which was developed ex professo for this application. WPA2 Personal [65] has been selected as the security mechanism considering data sensitiveness. Figure 9 shows the implemented code in Arduino IDE for alarm generation. Alarm management is performed from each device, sending an e-mail to one or more addresses, identifying the establishment and the refrigerator where the incident occurred.

The operating logic at the level of alarm generation is based on the definition of an admissible temperature range and an offset time. This avoids the generation of false alarms caused by the defrost cycles of the condensing batteries.

### 3.2. System Scaling

The proposed solution allows for easy scalability at both individual store and store chain level, either in chilling (temperatures about 0 °C) or freezing (temperatures under −18 °C) [66].

When applying the system to a whole store chain, the need arises to carry out the initial configuration of the boards and possible changes in remote mode. For this purpose, a LAMP (Linux + Apache + MySQL + PHP) server has been developed, hosted on a Raspberry Pi computer.

Each device connects daily to the DataBase and updates the values of: Supermarket, number of sensors, sensor ID, device status, maximum temperature per sensor, minimum temperature per sensor, time out of range for alarm generation, addresses to which alarm email is sent, ThingSpeak channel ID, write API key (WAP) of the ThingSpeak channel, read ThingSpeak channel API key (RAK), and ThingSpeak channel field.

**Figure 10:**
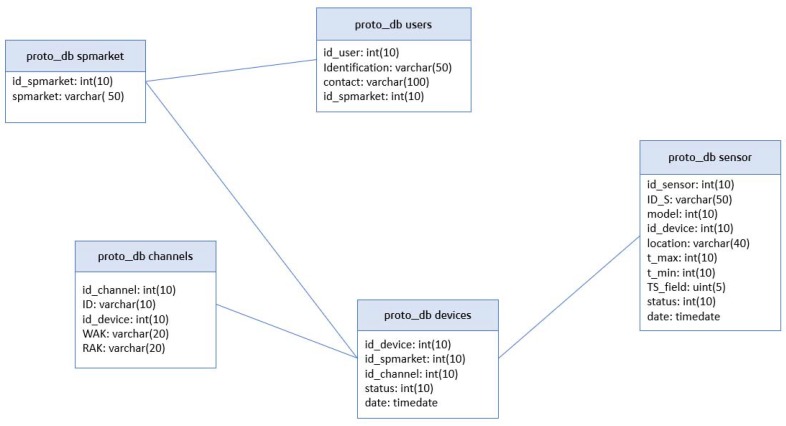
Database structure.

The database consists of five components (Figure 10): (i) *proto_db spmarket* refers to the name and number id of a supermarket; (ii) *proto_db users* registers the email address of maintenance managers with an identifying number (each email address is associated with a store); (iii) *proto_db channels* records the ThingSpeak channels to which each board must send temperatures; and (iv) *proto_db devices* collects data from the boards, its identifying numbers and operating status (error code: 0 if operating correctly). In addition, each device is associated with a channel and a store; (v) *proto_db sensors* collects the sensor data (identifier, maximum and minimum temperature, model, and operating status) associated with a device.

The standard version of ThingSpeak, which provides up to 250 channels of eight fields, that is up to 2000 variables that can be updated every second, is suitable for the deployment of the project in all the establishments of the chain. The maximum number of data per year is 33,000,000.

## 4. Experimental Scenario

The solution was implemented in a 372 m^2^ (public zone) supermarket, located in Cordoba (Spain). It offers different types of products requiring refrigeration, placed in refrigeration equipment whose characteristics are shown in Table 6.

**Figure 11:**
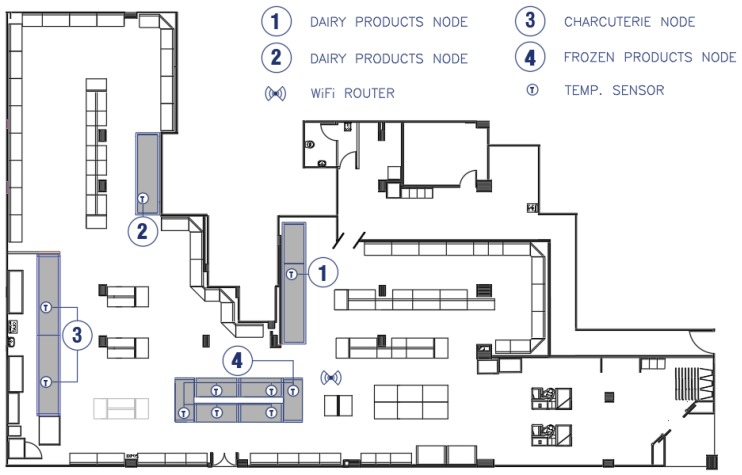
Layout of the installed elements in the store.

The supermarket has six autonomous freezers, two dairy display cabinets, and two vitrines, one for charcuterie and one for meat. The sensors of the freezers were connected to the same board. There was one sensor for the vitrines connected to another board and for the dairy cabinets there were two sensors connected to an individual board each. Therefore, nine temperature sensors connected to four electronic boards were installed, located as shown in Figure 11.

**Figure 12:**
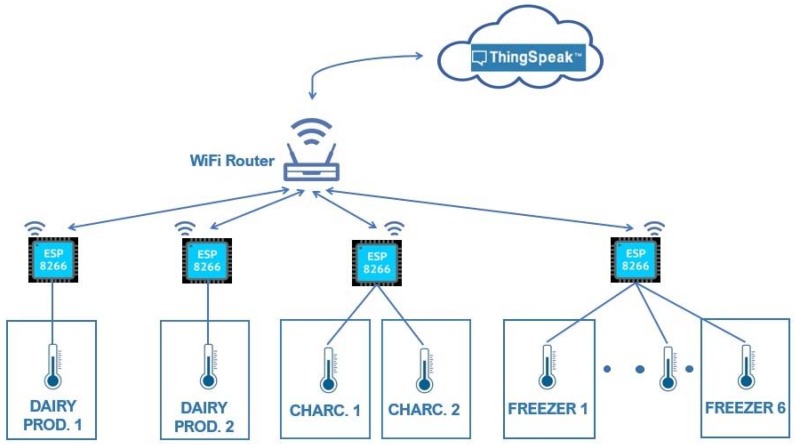
Definitive architecture for the adopted solution.

Figure 12 details the design of the proposed technological solution.

### 4.1. Generated Data

The temperature data reached the platform at two-minute intervals per sensor. Table 7 shows the data set generated by the global solution (3.4 million data). The first three nodes (chilling cabinets and vitrines) received information from one sensor each. Node 4 received information from six sensors, one for each freezer.

**Figure 13:**
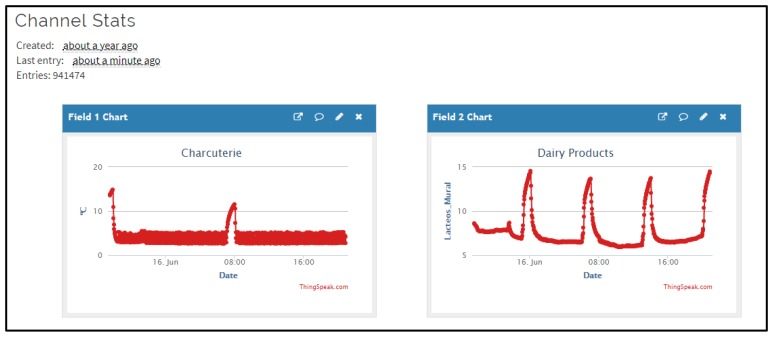
ThingSpeak visualization of temperature records associated to different display cabinets.

Figure 13 shows an example of the operation of two display cabinets for a specific day as offered by the ThingSpeak platform. It can also be displayed in an app (ThingChart) for smartphones. These charts were developed by programming in Matlab through the ThingSpeak platform.

### 4.2. System Evaluation

The proposed system in the selected store was launched on 25 January 2018 and is currently functioning. To evaluate the performance of the system, a comprehensive analysis of the solution was conducted from implementation to 15 August 2019. System evaluation focused on three aspects: Functionality in the generation of alarms, performance of the archiquecture system, and cost.

**Figure 14:**
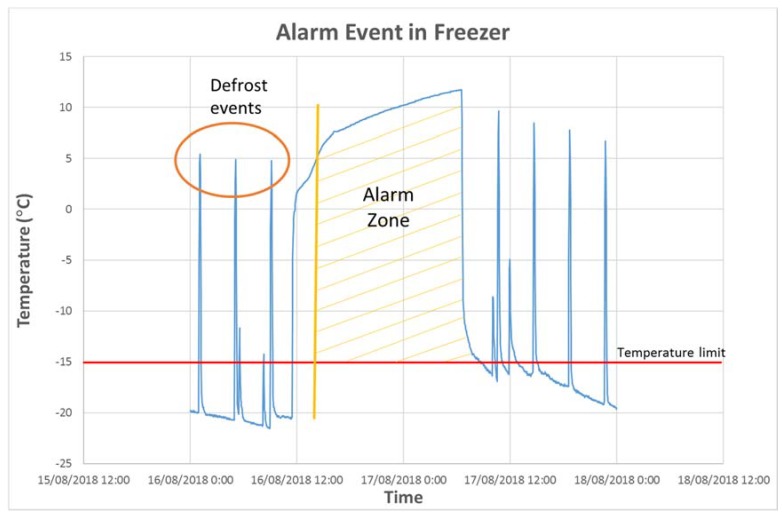
Example of alarm event.

Concerning the functionality scope, during the system operation, seven alarms were generated allowing action to be taken on the equipment before the product was to perish due to a cold chain failure. Figure 14 shows an example of an alarm event in a freezer. In this case, an alarm was detected when temperature limit (−15 °C) was exceeded for a set time (60 min). This value is configurable for each type of cabinet.

For the event shown in Figure 14, the detection of this alarm enabled the removal of the product, preventing losses of 1500 € (Table 6). The system used to send alarms via e-mail is decentralised and each device manages the alarms associated with the monitored equipment. Moreover, the system generates a complete report (temperature time series, in csv files and graphics as Figure 13) substituting information obtained from classical thermographs used for health services inspection [9]. Systems such as the one proposed, which reduce product waste, solve one of the main economic problems of the retail sector at European level [16].

**Figure 15:**
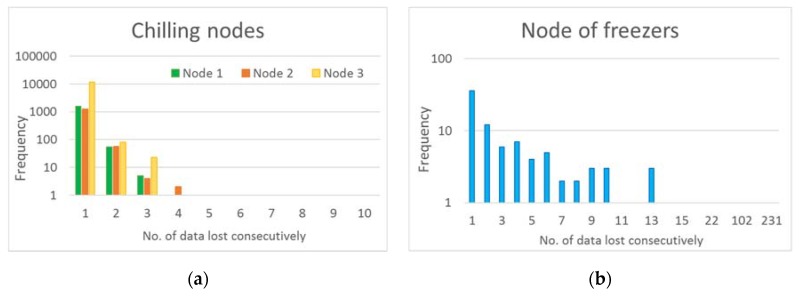
Data lost consecutively in chilling nodes (**a**) and the node of freezers (**b**).

With regards to system performance, the evaluation focused on the three levels of the solution architecture. In the area of measurement, there was only one specific failure registered in the reading from one sensor of freezer one. A reading failure indicates a measurement outside the range, i.e., above 40 °C or below −50 °C. When that happens, the data sent is 100, so it is easy to identify it among the other measurements. With regard to the control and processing area, no failure occurred in the electronic boards used and there was no need of replacement either. However, in the communications section, incidents recorded are worthy to be discussed. As shown in Table 7, although some data from all four nodes were lost, the percentages of data lost were negligible in three cases. Figure 15 analyzes lost data with more details. The figure shows the amount of data that was lost consecutively. In all nodes, it is observed that the higher frequency is associated with the loss of one single data, indicating, in terms of temperature control, that information was received every four minutes instead of every two minutes. Therefore, most of the lost data did not result in an important interruption of temperature control.

The failures can be attributed to three causes: (i) unavailability of the IoT (ThingSpeak) platform, (ii) internet connection error, and (iii) loss of connection of each node to the local Wi-Fi network. Since at the exact moment of data loss of a particular node, no simultaneous loss of the other nodes was recorded, the failures cannot be attributed to causes i and ii (these causes could also be a power failure). Therefore, all data losses were due to problems of connection of each node with the Wi-Fi. In this way, it could be expected that nodes 2 and 3, farther away from the router (see Figure 11), present a greater loss of sent data. However, results show that only node 3 had the greatest loss of data. This may be because node 3 is inside the cabinet, while node 2 is located above the cabinet. Being inside the cabinet may attenuate coverage, which may explain the greater amount of missing data associated with node 3.

Being able to continuously monitor the temperature addresses current challenges in improving quality control and transparency [6]. These changes are particularly relevant in areas with extreme climates, such as southern Spain, where previous studies had shown the importance of increasing temperature sampling frequency in retail establishments [9] and the need to introduce modernizations and technological improvements [10]. Furthermore, in this specific sector, the importance of having a time-temperature history has been highlighted as a critical aspect in perishable product price management [31].

Finally, concerning evaluation in terms of cost, Table 8 shows the material costs used in the system for the selected store, as well as the manpower required.

As shown in Table 8 and Table 9, the total cost of the system (for a medium-sized store) can be considered an affordable solution. In developing countries, where most temperature abuses are reported, the cost of the proposed solution is a reasonable alternative, without losing robustness and efficiency [49]. This approach is also applicable to meet the requirements in terms of new technological developments offered to SMEs (*Small and medium*-*sized enterprises)* [29]. The reduced cost of each node justifies why the number of nodes was not optimized, which could even be one for the whole store (technologically feasible as shown in Section 3.1). The cost of wiring each sensor to the board is higher than providing a greater number of nodes next to each display case. In this sense, Tsang et al. [56] propose a system based on a Wi-Fi local network and a gateway (3G/4G/LTE) where nodes are powered by batteries. In the case presented, this option has been rejected due to the high energy consumption associated with Wi-Fi communication and performed by means of wiring, confirming the aforementioned. When comparing the proposed solution with the commercial ones, it highlights, besides the cost, the possibility of working with electrical power without batteries and the open-source philosophy that, as it has been underlined throughout the work, allows the scientific community to improve the current research proposal.

After two years of operation of the proposed solution, having evaluated the reliability of the sensors and microcontrollers, as well as the availability of the Internet connection and the IoT platform from the 3.4 million data generated, it has been demonstrated that this solution is applicable at industrial level, representing a novelty and a scientific step forward with respect to proposals based on prototypes that have not reflected their performance over a long period of time. The solution helps to highlight the potential of IoT-based technological approaches in terms of “independent networks, that have the capabilities of self-configuration and self-optimizing among many other characteristics”, as already pointed out by other authors [16], providing an example of IoT application in the retail sector, where the adoption of this type of technology is still at an incipient stage, as other works remarked [24].

Additionally, distinctive aspects of the proposed solution, such as the use of open source hardware and software and its scalability and compatibility for integration into broader monitoring systems, are perfectly consistent with the guidelines of inter-organizational cooperation, transparency, and information-sharing argued by several authors as essential factors to improving efficiency, minimizing food waste, increasing product quality, improving sustainability factors, and the overall strategic position of food supply chains [67,68].

## 5. Conclusions

This work proposes the integration of several open-source hardware and software systems applied to the control of retail cold chain maintenance. Cost-effectiveness and reliability results confirm a very competitive IoT solution within the market. This competitiveness increases even more when considering the system as an open programming system, so that the architecture of the solution can be used to monitor and produce alarms in other relevant variables (door opening-closing, consumer presence time in front of the exhibitor, lighting, and energy consumption).

The operation and applicability of the solution has been evidenced by the evaluation performed on 3.4 million data obtained over twenty months. The proposed solution improves cold chain management control and monitoring in the retail sector, leading to better-quality standards traceability. As a result, this system simplifies the control tasks of inspectors or auditors (reports are automatically generated instead of using thermographs and dataloggers). Furthermore, this affordable solution enables an improvement in the corporate image for food retail companies which shows transparency in the way they control temperature and assure food safety, one of the major problems facing humanity.

The solution was installed in one establishment; however, expansion to the full network of establishments (75) is already in progress so the outstanding benefits will be extended to more stores.

In future works, the deployment of this solution over a prolonged period of time will provide a large volume of data leading to new approaches to data management (big data, trend analysis, adaptive models, neural networks, and deep learning) aimed at predictive maintenance, equipment comparison, and price management. A specific interface might also be designed to overcome the limitation that the generic one of Thingspeak could present for an efficient management of multiple establishments.

## Figures and Tables

**Table 1:** Short range wireless networks communication technology characteristics.

Parameter	Wi-Fi	Bluetooth	Zigbee	RFID	NFC
Standard	IEEE 802.11 a,b,g,n	802.15.1	802.15.4	Several standards	ISO/IEC 13157
Frequency	2.4 GHz	2.4 GHz	868/915 MHz, 2.4 GHz	13.56MHz	13.56MHz
Data rate	2–54 Mbps	1–24 Mbps	20-250 kbps	423 kbps	424 kbps
Transmission Range	20–100 m	8–10 m	10–20 m	1 m	0.1 m
Topology	Star	Star	Tree, star, mesh	Point to point	Point to point
Energy consumption	High	Medium	Low	Low	Low
Cost	Low	Low	Low	Low	Low

**Table 2:** Features of the electronic boards considered.

	NodeMCU	Heltec Wi-Fi LoRa 32 V2	Arduino MKR1000	Pycom WiPi 3.0	ELECTRODRAGON ESP RELAY
Microcontroller	ESP8266	ESP32	SAMD21	ESP32	ESP8266
Programming	Arduino IDE Compatibility	Arduino IDE Compatibility	Arduino IDE Compatibility	MicroPython	Arduino IDE Compatibility
ROM	32 kB	448 kB	256 kB	448 kB	32 kB
RAM	32 kB	520 kB	32 kB	520 kB	32 kB
Logic level	3.3 V	3.3 V	3.3 V	3.3 V	3.3 V
Analog input	10	18	5	18	1
Digital I/O	20	28	2	23	5
Other Features	Wi-Fi, BLE	Wi-Fi, BLE, OLED display	Wi-Fi	Wi-Fi, BLE,	230 Vac POWERING, Wi-Fi
Price (2019)	30 €	12 €	45 €	35 €	6 €

**Table 3:** Resistive and electronic temperature sensor characteristics.

Parameter	Thermistor	RTD	Thermocouple	Silicon Sensors	Infrared (IR) Pyrometers
Type	Resistive	Resistive	Electronic	Electronic	Electronic
Range (°C)	−100 to 300	−200 to 600	−200 to 2300	−271 to 200	−18 to 538
Accuracy	High	High	Medium	Medium	High
Size	Small	Large	Small	Small	High
Linearity	Nonlinear	Linear	Nonlinear	Linear	Nonlinear
Cost	Low	High	Low	Low	High

**Table 4:** Open-source equipment for the proposed solution.

Equipment	Hardware/Software	Functionality
Electrodragon ESP Relay	Hardware	Controlling and processing temperatures
IDE Arduino	Software	Programming temperatures alarms, communication, information placement
Raspberry Pi	Hardware	Server hosting
LAMP (Linux + Apache + MySQL + PHP) server	Software	Updating values for DataBase

**Table 5:** DS18B20 sensor characteristics [62].

Parameter	DS18B20	Units
Supply Voltage	3–5.5	V
Resolution	9–12	bits
Measure Range	−55 to +125	°C
Thermometer error −10 °C to 85 °C	± 0.5	°C
Thermometer error −30 °C to −10 °C	± 1	°C
Stand by current	750	nA
Active current	1	mA
Communication protocol	1 wire	
Cost (stainless steel shell) (2019)	2	€

**Table 6:** Refrigerated cabinets in the pilot shop.

.	Dairy Products	Charcuterie/Meat	Frozen Products
Type	Display cabinet	Vitrine	Cabin
Manufacturer	KOXKA	KOXKA	COSTAN
Model	SAMFL-3	SV-6/CI	EC26 1850
Units	2	2	6
Set Point (°C)	2	2	−18
Client access	Yes	No	Yes
Net volume (dm^3^)			600
Dimensions L×W×H (mm)	5800 × 1130 × 2000 3900 × 1130 × 2000	3800 × 1100 × 1200	
Stored product ($)	2200 1480	2800	1500

**Table 7:** Records of generated data.

	Node 1	Node 2	Node 3	Node 4
Total amount of generated data	339,876	339,867	339,739	2,365,602
Sent data	338,470	338,181	327,973	2,359,650
Lost data	1406	1686	11,766	5952
% of lost data	0.5	0.4	3.5	0.25

**Table 8:** Implemented solution cost.

Concept	Unit Cost ($)	Units	Total ($)
	MATERIAL COST		
ELECTRODRAGON ESP8266	6.85	4.00	27.40
Temperature Sensor DS18B20 (Waterproof)	2.10	10.00	21.00
Wi-Fi Router (Existing)	0.00	1.00	0.00
Extension wires for sensors (meters)	0.80	12.00	9.60
			**58.00**
	LABOUR COST		
Labour (hours)	30	7.5	**225**

**Table 9:** Features of commercial solutions versus research proposal.

Commercial Systems	Communications	Power Supply	Measure Unit Cost ($)	Gateway Cost ($)	Platform Cost ($/Year)	Philosophy
JRI MySyrius	RF/Ethernet	Battery	155.5	384.5	1000 up to 500 units	Proprietary
JRI MySyrius	LoRa/Ethernet	Battery	182.2	684.5	1000 up to 500 units	Proprietary
SEEMOTO	Bluetooth/Ethernet	Battery	167–222	no need	133.3 per unit	Proprietary
TESTO Saveris 2	Wi-Fi/Ethernet	Battery	140	no need	17.8 per unit	Proprietary
THINGPARK	LoraWan	Battery	143.3	193.3	38.9 per unit	Proprietary
COMPLIAN-CEMATE Plus	LoRa/Ethernet	Battery			1555 per five units	Proprietary
NTT Docomo USA		Battery			1189 per two units	Proprietary
AKOdata IoT	NB-IoT	Battery	617.8	no need	Included for two years	Proprietary
**Proposed solution**	**Wi-Fi/Ethernet**	**65–265 Vac**	**10**	**no need**	**666.7 up to 310 units**	**Open-source**
